# Detection of Aromatic Hydrocarbons in Aqueous Solutions Using Quartz Tuning Fork Sensors Modified with Calix[4]arene Methoxy Ester Self-Assembled Monolayers: Experimental and Density Functional Theory Study

**DOI:** 10.3390/molecules28196808

**Published:** 2023-09-26

**Authors:** Shofiur Rahman, Mahmoud A. Al-Gawati, Fatimah S. Alfaifi, Wadha Khalaf Alenazi, Nahed Alarifi, Hamad Albrithen, Abdullah N. Alodhayb, Paris E. Georghiou

**Affiliations:** 1 Biological and Environmental Sensing Research Unit, King Abdullah Institute for Nanotechnology, King Saud University, P.O. Box 2455, Riyadh 11451, Saudi Arabia; 2 Department of Physics and Astronomy, College of Science, King Saud University, P.O. Box 2455, Riyadh 11451, Saudi Arabia; falfaifi@ksu.edu.sa (F.S.A.);; 3 Department of Chemistry, Memorial University of Newfoundland, St. John’s, NL A1C 5S7, Canada

**Keywords:** quartz tuning fork, calix[4]arene, self-assembled monolayer, aromatic hydrocarbons, density functional theory

## Abstract

Quartz tuning forks (QTFs), which were coated with gold and with self-assembled monolayers (SAM) of a lower-rim functionalized calix[4]arene methoxy ester (CME), were used for the detection of benzene, toluene, and ethylbenzene in water samples. The QTF device was tested by measuring the respective frequency shifts obtained using small (100 µL) samples of aqueous benzene, toluene, and ethylbenzene at four different concentrations (10^−12^, 10^−10^, 10^−8^, and 10^−6^ M). The QTFs had lower limits of detection for all three aromatic hydrocarbons in the 10^−14^ M range, with the highest resonance frequency shifts (±5%) being shown for the corresponding 10^−6^ M solutions in the following order: benzene (199 Hz) > toluene (191 Hz) > ethylbenzene (149 Hz). The frequency shifts measured with the QTFs relative to that in deionized water were inversely proportional to the concentration/mass of the analytes. Insights into the effects of the alkyl groups of the aromatic hydrocarbons on the electronic interaction energies for their hypothetical 1:1 supramolecular host–guest binding with the CME sensing layer were obtained through density functional theory (DFT) calculations of the electronic interaction energies (ΔIEs) using B3LYP-D3/GenECP with a mixed basis set: LANL2DZ and 6-311++g(d,p), CAM-B3LYP/LANL2DZ, and PBE/LANL2DZ. The magnitudes of the ΔIEs were in the following order: [Au4-CME⊃[benzene] > [Au4-CME]⊃[toluene] > [Au4-CME]⊃[ethylbenzene]. The gas-phase BSSE-uncorrected ΔIE values for these complexes were higher, with values of −96.86, −87.80, and −79.33 kJ mol^−1^, respectively, and −86.39, −77.23, and −67.63 kJ mol^−1^, respectively, for the corresponding BSSE-corrected values using B3LYP-D3/GenECP with LANL2dZ and 6-311++g(d,p). The computational findings strongly support the experimental results, revealing the same trend in the ΔIEs for the proposed hypothetical binding modes between the tested analytes with the CME SAMs on the Au-QTF sensing surfaces.

## 1. Introduction

The aromatic hydrocarbons benzene, toluene, ethylbenzene, and xylene, which are collectively and commonly referred to as BTEX, are components of gasoline hydrocarbons. They are also part of the general grouping of volatile organic compounds (VOCs), which are found ubiquitously in our global environment [1]. BTEX compounds are released into the environment by a variety of different sources, which Yu et al. have classified as either being pyrogenic or petrogenic or arising from processed products [2]. As examples, BTEX compounds are released into the atmosphere as a result of the incomplete combustion of gasoline by motor vehicles and also from the combustion of organic materials or biomass, as well as other fossil fuels, including coal and crude oil. All of these are examples of pyrogenic sources. Oil seepages, which can occur naturally or from accidental oil spills, also discharge numerous VOCs, including BTEX [1,2], into the environment. These and other less-volatile polycyclic aromatic hydrocarbons (PAHs) can pose a significant threat both to the environment and directly to human health [3] in many parts of the world. These effects have been well researched and documented in the scientific literature [1,2,3,4,5,6]. Furthermore, crude oil processing has been reported to contribute up to 16% of hydrocarbon VOC emissions [7]. The preceding are all examples of petrogenic sources. In addition to being released into the atmosphere, many of these compounds appear in soil and potable water supplies, through the contamination of either aquifers or the water supply, e.g., lakes, rivers, or dams themselves. Although they are generally considered to be water-immiscible, the reported data on the water solubilities of benzene, toluene, and ethylbenzene are 1.80 g/L [8], 0.519 g/L [9], and 0.015 g/100 mL [10], respectively. These levels are significantly higher than the U.S. Environmental Protection Agency (EPA)’s maximum permissible levels of 5 µg/L, 1 mg/L, and 700 µg/L, respectively [11]. As an example of petrogenic environmental water contamination, Adeniran et al. provided a comprehensive review of groundwater contamination by crude oil spillages [12]. On the other hand, in addition to the fact that BTEX compounds enter the environment through the sources referred to above, other sources include industrial activities, and BTEXs have been found to be common groundwater contaminants at 1 to 3 μg/L [13]. This is because they are the most-frequently produced petrochemical intermediate chemicals, which, in addition to their use in gasoline, are also widely used as solvents in rubber, chemical, coating, dyeing, glue, printing, pesticides, paints, fuel additives, pharmaceutical industries, etc. These are examples of the third and “processed” sources of BTEX referred to by Yu et al [2]. Benzene is essential for producing products such as polystyrene from its ethylbenzene derivative, detergents, medications, paints, and pesticides, but it is particularly toxic by itself [14]. Ethylbenzene’s dehydrogenation into styrene contributes to 85% of the total of styrene manufactured [15]. Toluene is extensively used in paint products [2], but it is not as toxic as benzene and ethylbenzene, so it is used indoors, where, initially, relatively high air levels can be measured [16].

Unfortunately, the concentrations of BTEX in the environment have increased significantly over the years, due to the main sources described above. This pollution has led to aquifer, groundwater, and surface-water contamination [2], which have also had a significant impact on marine organisms, even at low levels. Therefore, developing efficient, high-performance, and environmentally friendly techniques that allow the ultrasensitive and precise detection of BTEX on small samples without requiring significant preparation times is highly desirable.

Many analytical techniques have been used to quantify the concentration of benzene, toluene, and ethylbenzene in water at trace levels, including gas chromatography–mass spectrometry (GC-MS) [17], headspace-gas chromatography–mass spectrometry (HSGC-MS) [18], high-performance liquid chromatography (HPLC) [19], UV–laser-induced fluorescence spectroscopy [20], and polymer-based quartz crystal microbalance (QCM) sensors [21]. Although many of these methods have excellent detection limits and are capable of multi-element analysis, they also have some limitations in relation to the precise detection of trace amounts of aromatic compounds, such as benzene, toluene, and ethylbenzene. These limitations include expensive equipment facilities, knowledgeable and skilled personnel, and time-consuming and complex sample preparation.

Microcantilever sensors [22] and quartz tuning fork (QTF) sensors [23] based on micro-electromechanical systems (MEMSs) have been successfully used for detecting trace amounts of chemical species in aqueous media. These sensors can be used for various purposes, such as environmental monitoring, chemical warfare and the detection of explosives, and also, in medical research. It is important to note that each method has its own advantages and disadvantages, including differences in sensitivity, selectivity, cost, complexity, and the required sample preparation. The choice of method depends on the specific needs of the analysis, such as having a suitable sensing layer, the desired detection limit, the sample matrix, and available resources.

Calixarenes have been extensively studied for their versatile properties as stable building blocks upon which functional groups can be fine-tuned to form supramolecular complexes with diverse analytes in both aqueous and organic solutions [24]. Zeybek et al. reported the use of a QCM sensor coated with a lower-rim substituted diamide calix[4]arene for the detection of gas-phase benzene, toluene and *m*-xylene [25]. We previously employed a lower- and upper-rim-functionalized calix[4]arene methoxy ester (CME) as a sensing layer on gold (Au)-coated microcantilevers to detect metal ions and their counterions in aqueous solutions [26,27]. The study presented herein extends the application of the same calixarene as a sensing layer on Au-coated QTFs to detect the aromatic hydrocarbons benzene, toluene, and ethylbenzene in dilute water solutions. The experimental results were augmented with a quantum chemical density functional theory (DFT) study to provide an insight into the nature of the hypothetical interactions between the individual aromatic hydrocarbons and the CME receptor molecule.

## 2. Results

### 2.1. Resonance Frequency Measurements

**Figure 1:**
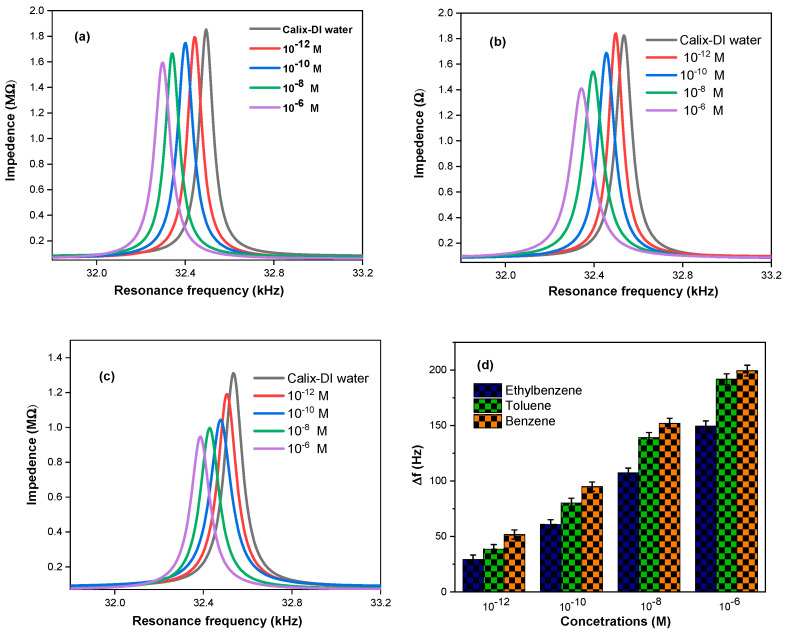
Comparison of the resonance frequency responses of the CME-functionalized Au-coated QTFs with the different concentrations (10^−12^ M to 10^−6^ M) of each of the aqueous solutions of (**a**) benzene, (**b**) toluene, and (**c**) ethylbenzene; (**d**) is a summary histogram showing the relative resonance frequency shifts (Δf) +/− ~5% of the three aromatic hydrocarbons tested.

Resonance frequency measurements obtained with Au-coated QTFs functionalized with THE calix[4]arene methoxy ester sensing layer (Figure 1) were used to quantitatively and qualitatively detect the presence of benzene, toluene, and ethylbenzene in water solutions. The resonance frequency of the QTF was scanned from 30.9 to 35.0 kHz using the software control of the system previously described by us [22]. Each cycle was completed in a maximum of approximately 30 s. The resonance frequency shifts were calculated using Equation (1) [22,23], and the results are shown in Figure 1d.

(1) $$\Delta f=f_{ref}-f_{a}$$

where *f_ref_* and *f_a_* are the resonance frequencies of the distilled water control and the tested analytes at the 10^−12^, 10^−10^, 10^−8^, and 10^−6^ M concentrations.

Figure 1a–d show the comparison of the resonance frequency responses of the coated QTFs when immersed in the different concentrations (10^−12^ to 10^−6^ M) of the individual analytes benzene, toluene, and ethylbenzene in deionized water (DI) solutions. Figure 1a shows that the largest resonance frequency shift, Δf = 198 Hz, is the decrease of the resonance frequencies from 32,494 Hz to 32,296 Hz measured after immersion in the 10^−6^ M solution of benzene in DI. Smaller, linear shifts were recorded for the more-diluted solutions. Resonance frequency decreases were also observed for the other analyte solutions, as shown in Figure 1b,c and a detailed discussion follows in Section 3. The highest resonance frequency shift for the corresponding 10^−6^ M solutions was in the following order: benzene (198 Hz) > toluene (191 Hz) > ethylbenzene (149 Hz). The responses of the Au-coated QTFs without the CME SAMs showed very small frequency shifts in the following order: benzene (35 Hz) > toluene (34 Hz) > ethylbenzene (31 Hz), as shown in Figure S3.

### 2.2. Quantum Chemical DFT Calculations

To gain a better understanding of the interaction between the CME with each of the three analytes, quantum chemical density functional theory (DFT) calculations were conducted using *Gaussian 16, Revision C.01* [28], with the CAM-B3LYP [29] and PBE [30] functionals with the LANL2DZ basis set [31], in the gas phase and water solvent system. Furthermore, the B3LYP-D3/GenECP and LanL2DZ [31] basis sets were used for Au, and the 6-311++g(d,p) basis set was used for all other atoms. The electronic interaction energy (ΔE_int_ kJ mol^−1^) values were calculated using Equations (2) and (3) for the components of the modeled hypothetical 1:1 supramolecular complexes formed by the Au-bonded receptor CME with the respective analytes. These results are discussed in further detail in the Section 3.2.

ΔE_int_ for CME⊃Analyte = E_[CME]⊃[Analyte]_ − (E_[CME]_ + E_[Analyte]_) (2)

ΔE_int_ for [Au4-CME]⊃Analyte = E_[Au4-CME]⊃[Analyte]_ − (E_[CME]_ + E_[Analyte]_) (3)

where E_[CME]⊃[Anayte]_ = optimized energy of the CME complex(es) with the specific analyte; E_[Au4-CME]⊃[Analyte]_ = optimized energy of the Au4-CME 1:1 supramolecular complex(es) with the specific analyte; E_[Au4-CME]_ = optimized electronic energy of the Au-receptor Au4-CME; E_[CME]_ = optimized electronic energy of the free receptor CME; E_[Analyte]_ = optimized electronic energy of the specific benzene, toluene. or ethylbenzene analyte. 

The DFT-calculated electronic binding interaction energies (ΔIE kJ mol^−1^) are summarized in Table 1, Table 2 and Table 3. The negative ΔIE values represent stronger interactions between the CME receptor with the analytes, which correlates with our experimental results. Table 1 shows the electronic binding interaction energies (ΔIE kJ mol^−1^) with uncorrected BSSE values with the CAM-B3LYP/LAN2DZ and PBE/LANL2DZ functionals in the water solvent system. The calculated gas phase and water solvent system ΔIEs with the CAM-B3LYP/LAN2DZ and PBE/LANL2DZ functionals were in the following order: [CME⊃[Benzene] > [CME]⊃[Toluene] > [CME]⊃[Ethylbenzene] −51.99, −45.66, and −41.92 kJ mol^−1^, respectively, in the gas phase and −32.12, −31.28, and −30.5 kJ mol^−1^, respectively, in the water solvent system. A similar trend was also observed with PBE/LANL2DZ in the gas phase and the water solvent system. The results from both methods were consistent. The DFT-calculated results strongly supported the experimentally obtained results.

## 3. Discussion

### 3.1. Analysis of the Resonance Frequency Measurements

**Figure 2:**
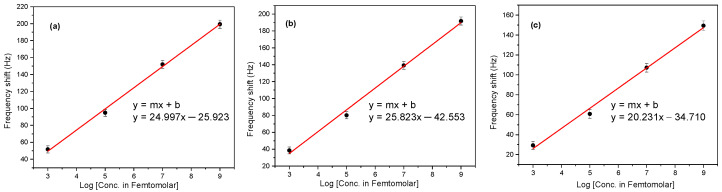
Comparison of the LODs of the CME-functionalized Au-coated QTFs with the different concentrations (10^−12^ M to 10^−6^ M) of each of the aqueous aromatic hydrocarbon solutions: (**a**) benzene, (**b**) toluene, and (**c**) ethylbenzene. These black dots reflect error bars of approx 5%.

The Au-coated QTFs were functionalized with CME as previously described (Figure 1). The resonance frequency changes that were measured at increasing concentrations of the analytes in DI showed that the methodology was sufficiently sensitive to detect concentrations of the three aqueous analyte solutions as low as 10^−12^ M. All experiments were conducted in triplicate, separately, at four different concentrations (10^−12^, 10^−10^, 10^−8^, and 10^−6^ M) for each of the analytes, benzene, toluene, and ethylbenzene. All replicates were within SD of +/− ~5%. QTF sensor measurements were performed between each solute measurement with Milli-Q DI water as control experiments. Furthermore, measurements were conducted using Au-coated QTFs without any of the sensing CME to confirm that there were no frequency changes, thus indicating that no interactions of the analytes occurred with the Au-coated QTFs’ surfaces alone. Figure 1a shows the largest resonance frequency shift, Δf = 199 Hz, which was measured with the CME-functionalized QTF in aqueous 10^−6^ M benzene solution. Figure 1b,c show the corresponding largest resonance frequency shifts of Δf = 191 Hz and 149 Hz, for the aqueous 10^−6^ M toluene and aqueous ethylbenzene solutions, respectively. Figure 1d illustrates the resonance frequency shifts for the corresponding 10^−6^ to 10^−12^ M solutions of the three analytes, and in all cases, the order was as follows: benzene > toluene > ethylbenzene. Their limits of detection (LODs) [32,33,34] were 3.47 × 10^−14^ M, 9.43 × 10^−14^ M, and 1.75 × 10^−13^ M, respectively, as calculated from their respective linear fit curves by plotting the logarithm concentration versus the frequency shift using Equation (4) and shown in Figure 2.

(4) $$\mathrm{Log}\;(\mathrm{LOD})=\left(\frac{3.3\;\sigma }{m}\right)$$

where *σ* = standard deviation of the intercept and *m* = slope of the fit curve.

### 3.2. DFT Calculations

**Figure 3:**
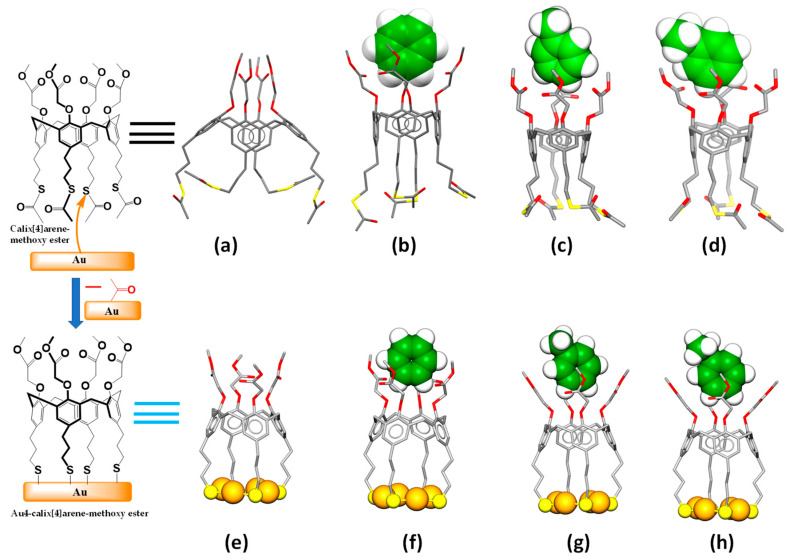
Geometry-optimized structures: (**a**) receptor molecule CME; (**e**) receptor Au4-CME; 1:1 binding modes of (**b**) CME⊃benzene, (**c**) CME⊃toluene, (**d**) CME⊃ethylbenzene, (**f**) Au4-CME⊃benzene, (**g**) Au4-CME⊃toluene, and (**h**) Au4-CME⊃ethylbenzene. Color code: carbon = gray (except benzene, toluene, and ethylbenzene carbon = green); gold = orange; oxygen = red; sulfur = yellow; hydrogen atoms = white for benzene, toluene, and ethylbenzene (other hydrogen atoms are omitted for clarity).

The initial molecular structures of all of the examined structures were drawn using *GaussView 6.0.16* [35], and all computations were conducted using *Gaussian 16*, *Revision C.01* [28]. Vibrational frequency analyses were conducted for each optimized structure to ensure that they each had a vibrational minimum energy and no imaginary frequencies. We hypothesized that by immersing a Au-coated QTF into the CME solution (at a concentration of 1.0 × 10^−6^ M) and then incubating it for 1 h, self-assembled monolayers (SAMs) of the CME would form on the gold surfaces of the QTFs. This would be due to the formation of the covalent S-Au bond and the acetyl (CH_3_C=O) fragments, as we previously showed [22,27,36,37]. A schematical illustration is shown on the left side in Figure 3. The methoxy ester group of the calixarene was further hypothesized to form supramolecular 1:1 complexes with each of the analytes, by electrostatic noncovalent “host–guest” interactions, as depicted in Figure 3a–d. To simplify the quantum chemical calculations and reduce the computation time, DFT calculations with the CAM-B3LYP/LANL2DZ and PBE/LANL2DZ functionals were also conducted using a single molecule of the CME bonded onto a gold cluster (Au4) for the complexation, as shown in Figure 3e–h, with the analytes, first in the gas phase and, then, also, in the water solvent system. Furthermore, the geometries of the hypothetical Au4-CME ([Au4-CME]) structures as their complexes with the analytes were conducted with B3LYPD3/GenECP and two different basis sets; the 6-311++g(d,p) basis set was used for the C, O, S, and H atoms (Equation (5)), and the LANL2DZ basis set was used for the Au atoms (Equation (6)), in both the gas phase and water solvent system. In order to access both basis set functions, the GenECP keyword was used. The Los Alamos National Laboratory 2 double ζ (LANL2DZ) effective-core-potential (ECP)-type basis set has been employed for heavy metal atoms, as is widely accepted [38], so employing the LANL2DZ for the gold (Au) was effective at reducing the computational resources. To avoid basis set superposition errors (BSSEs) [39,40,41,42] in the non-covalent interactions between receptor molecules and analytes, counterpoise (CP) corrections were made in the gas phase at the same level of theory. The binding interaction energies were calculated using the following equations:

ΔE_BSSE_ (CME⊃Analyte) = E(_[CME]⊃[Analyte]_) − E(_[CME]_) − E(_[Analyte]_) (5)

ΔE_BSSE_ ([Au4-CME]⊃Analyte) = E(_[Au4-CME]⊃[Analyte]_) − E(_[Au4-CME]_) − E(_[Analyte]_) (6)

where E(_[CME]⊃[Analyte]_) = optimized energy of the CME complex(es) with the specific analyte; E(_[Au4-CME]⊃[Analyte]_) = optimized energy of the receptor Au4-CME 1:1 complex(es) with each of the analytes; E(_[Au4-CME]_) = optimized electronic energy of the free receptor Au4-CME; E(_[CME]_) = optimized electronic energy of the free receptor CME; E(_[Analyte]_) = optimized electronic energy of the specific benzene, toluene, or ethylbenzene analyte.

The DFT-calculated electronic binding interaction energies (ΔIE kJ mol^−1^) are summarized in Table 1, Table 2 and Table 3. Table 1 shows the gas phase uncorrected BSSE and corrected BSSE electronic ΔIE values using CAM-B3LYP/LANL2DZ and PBE/LANL2DZ. The ΔIEs for the hypothetical 1:1 [Au4-CME]⊃analyte complexes were higher than the corresponding [CME⊃analyte] complexes. The 1:1 [Au4-CME]⊃[benzene], [Au4-CME]⊃[toluene] and [Au4-CME]⊃[ethylbenzene] complexes had the highest BSSE-uncorrected ΔIE values, −71.42, −65.40, and −54.39 kJ mol^−1^, respectively, and −48.60, −44.85, and −42.38 kJ mol^−1^, respectively, for the respective BSSE-corrected values with CAM-B3LYP/LANL2DZ in the gas phase. The same trends, albeit of lower energies, were also observed with PBE/LANL2DZ in the gas phase. Table 2 shows similar trends as for Table 1 for the electronic binding interaction energies with the CAM-B3LYP/LANL2DZ and PBE/LANL2DZ functionals and basis sets, in the water solvent system. The system with the former gave better results compared with PBE/LANL2DZ for both the gas phase and water solvent system. 

Table 3 shows the computed data for the gas phase and water solvent system for the [Au4-CME]⊃[benzene], [Au4-CME]⊃[toluene], and [Au4-CME]⊃[ethylbenzene] 1:1 complexes using B3LYP-D3/GenECP with LanL2DZ for Au and 6-311++g(d,p) for all other atoms. The highest BSSE-uncorrected ΔIE values were obtained with this functional/basis set: −96.86, −87.80, and −79.33 kJ mol^−1^, respectively, and −86.39, −77.23, and −67.63 kJ mol^−1^, respectively, for the corresponding BSSE-corrected values in the gas phase. The highest BSSE-uncorrected ΔIE values were −84.98, −75.25, and −66.28 kJ mol^−1^ in the water solvent. The DFT-calculated results strongly supported the experimentally obtained results, and although the same trends were seen with either functionals or basis sets, the B3LYP-D3/GenECP afforded the best results, i.e., gave the most-energetically favored electronic binding interaction energies. Uddin et al. reported the use of both CAM-B3LYP/LAN and PBE/LANL2DZ along with other levels of theory to identify variations with functionals and basis sets [43].

**Figure 4:**
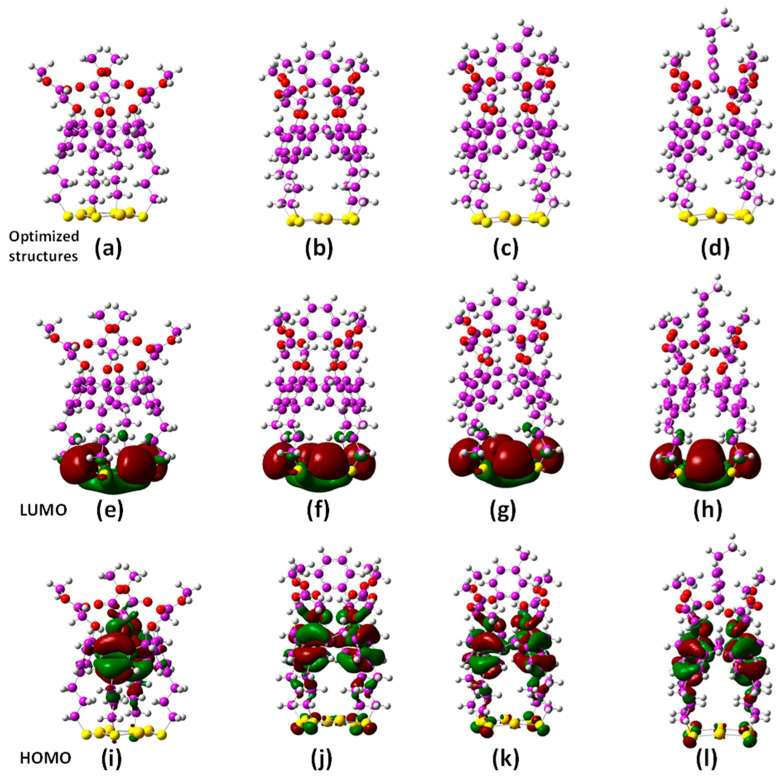
Geometry-optimized structures of Au4-CME and its 1:1 supramolecular complexes with benzene, toluene, and ethylbenzene (**a**–**d**) and their respective HOMO (**e**–**h**) and LUMO (**i**–**l**) FMOs. Color code: carbon = purple; gold = orange; oxygen = red; sulfur = yellow; hydrogen atoms = white.

To gain additional understanding of the nature of the interactions and stabilities between the free and Au-bound CME molecules with the three analytes, frontier molecular orbitals (FMOs) [44] were examined. These were based on the most-stable geometries generated for these molecules and their respective highest occupied molecular orbitals (HOMOs) and lowest unoccupied molecular orbitals (LUMOs), as shown in Figure 4.

Koopmans’ theorem considers that the energy levels of the HOMOs and LUMOs are related to their nucleophilic and electrophilic properties, respectively [45]. Large HOMO–LUMO gaps are indicative of the chemical species’ high stability and low reactivity. Table 4 shows that the HOMO–LUMO value for Au4-CME in the gas phase (7.050 eV) was lower than the water solvent system (7.319 eV). The lower HOMO–LUMO gap value of the receptor Au4-CME, therefore, understandably had a higher binding ability with the analytes in the gas phase compared with the water solvent system and the DFT-calculated binding energies as more negative for the gas phase. Presumably, the solvation of each molecule in the water solvent prevented their effective interactions. The solvation energy in water is more complex with hydrogen bonding having significant effects on the solvation energies in water. The HOMO–LUMO energy gaps for the complexes were in the following order: [Au4-CME⊃[benzene] > [Au4-CME]⊃[toluene] > [Au4-CME]⊃[ethylbenzene]. The HOMO–LUMO energy values for the CME, Au4-CME, each of the analytes, and their complexes are reported in Tables S1 and S2 (Supporting Information) and can be used to calculate a number of other significant and valuable quantum chemical properties such as the global hardness (η), global softness (S), electrophilicity index (ω), electronegativity (χ), and chemical potential (μ), which all measure chemical reactivity. These were generated using *GaussView 6.0*.16 [35]. The chemical potential (μ), hardness (η), softness (S), and global electrophilicity index (ω) of the receptor molecules CME, Au4-CME, analytes, and their complexes were calculated using the HOMO and LUMO energies. The formulas used for these calculations are given as follows: η = (E_LUMO_ − E_HOMO_)/2; μ = (E_HOMO_ − E_LUMO_)/2; S = 1/η and ω = μ^2^/2η.

## 4. Materials and Methods

### 4.1. Chemicals and Materials

Benzene, toluene, ethylbenzene, dichloromethane, and ethanol were procured from Sigma-Aldrich, St. Louis, MI, USA. The synthesis of the receptor sensing CME was previously reported by us [27]. All aqueous solutions of the aromatic compounds were prepared as saturated solutions using deionized (DI) water with a resistivity of 18.6 MΩ·cm. The pH of the deionized water was 6.82. The analyte solutions had a pH of 6.85. The Au-coated QTFs were purchased from Forien Inc., Edmonton, AB, Canada (https://www.fourien.com/). They were coated with Au using a vacuum evaporation method; the thickness of the gold coating was around 100 nm. The resonance frequency of the QTFs was 32.768 kHz; the spring constant was ~20 kN/m; the load capacitance was 12.5 pF. A 10^−6^ M solution of the CME was used to functionalize the Au-coated QTFs. 

### 4.2. Experimental Setup and Instrumentation

**Figure 5:**
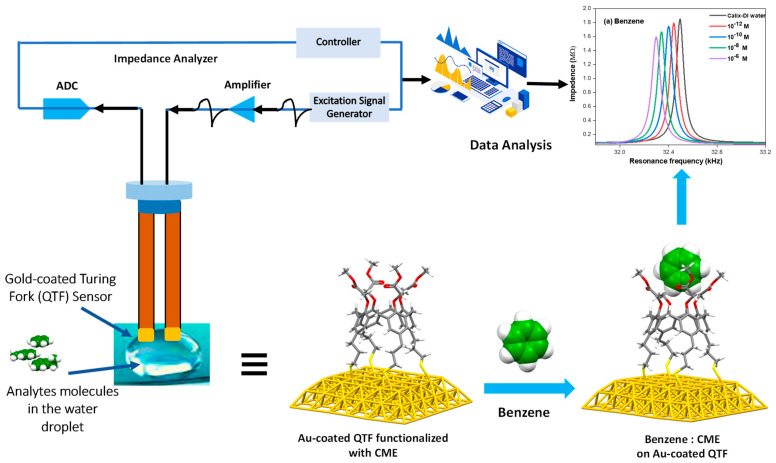
Schematical illustration of the QTF measurement system used and the Au-coated QTFs functionalized with CME and its hypothetical 1:1 supramolecular complex with benzene; (a) is a typical output showing the resonance frequency responses (in kHz) to the aqueous benzene solutions of different concentrations (10^−6^–10^−12^ M). Color code: carbon = gray (except benzene carbon = green); hydrogen = white; sulfur = yellow; oxygen = red; hydrogen atoms = white.

The frequencies of QTF resonance were measured with a Quester Q10 instrument made by Fourien Inc. The Quester Q10 contains an impedance analyzer that performs frequency sweeps and measures the impedance response’s real and imaginary components. To resonate the QTFs at specific frequencies, the proportional–integral–differential technique was used in this system. The system can keep the QTF at a fixed distance from any analyte solute when combined with a translation stage. The data collected were analyzed using MATLAB or the Origin Lab program. The system is illustrated in Figure 5, which includes a schematic of the methoxy ester CME SAM-functionalized Au-coated QTFs. A detailed description of the instrumental setup and software integration can be found in our previous study and elsewhere [22,23].

### 4.3. Experimental Methods

The gold-coated QTFs self-assembled monolayers (SAMs) of calix[4]arene were prepared by incubating the QTFs for 1 h in a solution of CME (1.0 × 10^−6^ M) at room temperature in a 1:9 dichloromethane:ethanol solvent. The QTFs were then washed three times with the same solvent mixture to remove any unbonded CME and were then dried using a nitrogen gas stream. The resonance frequency of the functionalized QTFs was measured in DI water for reference. Finally, the functionalized QTFs were exposed to different concentrations (10^−12^, 10^−10^, 10^−8^, and 10^−6^ M) of saturated aqueous (DI) solutions of the analytes (benzene, toluene, and ethylbenzene) for ten minutes, after which time, their resonance frequencies were measured to determine their sensitivity. To maintain consistent experimental conditions, the QTFs were directly immersed at a depth of 25 μm in a small droplet of a 100 μL volume of the respective analyte solutions.

## 5. Conclusions

In this study, we investigated the effectiveness of using a CME as a sensing receptor molecule for forming stable SAMs on the gold surfaces of QTFs. Our findings suggested that these SAMs have the potential for rapidly detecting the aromatic hydrocarbons benzene, toluene, and ethylbenzene in water samples, at very low analyte concentrations (10^−12^ M) and using small amounts of aqueous solutions (100 μL). Among the analytes that were tested, benzene had the highest effect on the QTF responses, with frequency shifts in the following order: benzene (199 Hz) > toluene (191 Hz) > ethylbenzene (149 Hz). This trend is consistent with the trends observed by Zeybek et al. for the vapor-phase BTX detections using a QCM with a thin-film layer of a calix[4]arene with a different functional group [25].

In our study, the limits of detection for benzene, toluene, and ethylbenzene were calculated to be 3.5 × 10^−14^ M, 9.4 × 10^−14^ M, and 1.8 × 10^−13^ M, respectively. Utilizing density functional theory (DFT), the interaction energies (ΔIEs) were calculated for the hypothetical 1:1 supramolecular complexes formed between the analytes within the methoxy ester moieties of the gold-bonded CME. The DFT-calculated ΔIE values for the 1:1 supramolecular analyte complexes with the Au-bonded Au4-CME receptor showed higher values compared to the free CME. Among the density functionals we tested, the B3LYPD3/GenECP functional with the effective core potential LANL2DZ for Au atoms and the 6-311++g(d,p) basis set for C, H, O, and S atoms showed the optimal binding interactions of the receptor CME with the analytes. The complexes of [Au4-CME]⊃[benzene], [Au4-CME]⊃[toluene], and [Au4-CME]⊃[ethylbenzene] had the highest BSSE-uncorrected and BSSE-corrected binding interaction (ΔIE) values, which were −96.86, 87.80, and −79.33 kJ mol^−1^, respectively, and −86.39, −77.23, and −67.63 kJ mol^−1^, respectively, for the respective BSSE-corrected values at the B3LYPD3/GenECP with the LANL2dZ and 6311++g(d,p) level of theory in the gas phase. Similar trends were observed when considering the hypothetical Au-CME complexes with the analytes, using both the CAM-B3LYP/LANL2DZ and LANL2DZ/PBE0 levels of theory in the gas phase and water solvent system. The DFT results showed that there was consistency in the ΔIE values obtained from the three above-mentioned methods. The ΔIE values of the selected DFT functionals along with the LANL2DZ and the 6-311++g(d,p) basis sets were in the following order: B3LYPD3 > CAM-B3LYP > PBE0. The results obtained through DFT calculations, considering both uncorrected and corrected BSSE values, strongly supported the experimental findings on the proposed binding modes between the analytes (benzene, toluene, and ethylbenzene) and the CME SAMs on the Au-QTF sensing surfaces.

## Figures and Tables

**Table 1:** Comparison of DFT-calculated electronic binding interaction energies (ΔIE kJ mol^−1^) for 1:1 complexes of the receptor CME with benzene, toluene, and ethylbenzene with the CAM-B3LYP/LANL2DZ and PBE/LANL2DZ functionals/basis sets, in the gas phase.

Complex	ΔIEs (kJ mol^−1^) of the Hypothetical 1:1 Supramolecular Structures of CME with Benzene, Toluene, and Ethylbenzene			
	CAM-B3LYP/LANL2DZ		PBE/LANL2DZ	
	Uncorrected BSSE	Corrected BSSE	Uncorrected BSSE	Corrected BSSE
[CME]⊃[Benzene]	−51.99	−34.19	−48.18	−31.08
[CME]⊃[Toluene]	−45.66	−30.66	−44.91	−29.95
[CME]⊃[Ethylbenzene]	−41.92	−25.06	−43.08	−26.13
[Au4-CME]⊃[Benzene]	−71.42	−48.60	−61.53	−44.18
[Au4-CME]⊃[Toluene]	−65.40	−44.85	−59.40	−42.57
[Au4-CME]⊃[Ethylbenzene]	−54.39	−42.38	−51.57	−41.14

**Table 2:** Comparison of DFT-calculated electronic binding interaction energies (ΔIE kJ mol^−1^) for 1:1 complexes of CME with benzene, toluene, and ethylbenzene with the CAM-B3LYP/LANL2DZ and PBE/LANL2DZ functionals/basis sets, in water solvent system.

Complex	ΔIEs (kJ mol^−1^) of the Hypothetical 1:1 Supramolecular Structures of CME with Benzene, Toluene, and Ethylbenzene	
	CAM-B3LYP/LANL2DZ	PBE/LANL2DZ
	Uncorrected BSSE	Uncorrected BSSE
[CME]⊃[Benzene]	−32.12	−29.77
[CME]⊃[Toluene]	−31.28	−28.80
[CME]⊃[Ethylbenzene]	−30.55	−28.34
[Au4-CME]⊃[Benzene]	−60.45	−53.31
[Au4-CME]⊃[Toluene]	−58.06	−50.93
[Au4-CME]⊃[Ethylbenzene]	−55.47	−47.19

**Table 3:** Comparison of DFT-calculated electronic binding interaction energies (ΔIE kJ mol^−1^) for the hypothetical 1:1 supramolecular complexes of Au4-CME with benzene, toluene, and ethylbenzene using B3LYP-D3/GenECP and LanL2DZ basis sets for Au and the 6-311++g(d,p) basis set for all other atoms in water solvent system.

Complex	ΔIEs (kJ mol^−1^) of the Hypothetical 1:1 Supramolecular Structures of CME with Benzene, Toluene, and Ethylbenzene		
	Gas Phase		Water Solvent
	Uncorrected BSSE	Corrected BSSE	Uncorrected BSSE
[Au4-CME]⊃[Benzene]	−96.86	−86.39	−84.98
[Au4-CME]⊃[Toluene]	−87.80	−77.23	−75.25
[Au4-CME]⊃[Ethylbenzene]	−79.33	−67.63	−66.28

**Table 4:** DFT-calculated global scalar properties of the receptor molecule Au4-CME and its complexes with the analytes at the CAM-B3LYP/LANL2DZ level of theory in the gas phase and water solvent system.

	HOMO Energy eV	LUMO Energy eV	H–L Gap eV	Ionization Potential (IP) eV	Electron Affinity (EA) eV	Electronegativity (χ) eV	Chemical Potential (μ) eV	Hardness (η) eV	Softness (*S*) eV	Electrophilicity Index (ω) eV
Gas phase										
Au4-Calix[4]arene	−7.230	−0.181	7.050	7.230	0.181	3.706	−3.706	3.525	0.284	1.948
Au4-CME⊃benzene	−7.337	−0.202	7.135	7.337	0.202	3.770	−3.770	3.568	0.280	1.992
Au4-CME⊃toluene	−7.325	−0.196	7.129	7.325	0.196	3.761	−3.761	3.564	0.281	1.984
Au4-CME⊃ethylbenzene	−7.321	−0.195	7.126	7.321	0.195	3.758	−3.758	3.563	0.281	1.982
**Water solvent system**										
Au4-CME	−7.526	−0.206	7.319	7.526	0.206	3.866	−3.866	3.660	0.273	2.042
Au4-CME⊃benzene	−7.625	−0.160	7.465	7.625	0.160	3.892	−3.892	3.732	0.268	2.030
Au4-CME⊃toluene	−7.622	−0.159	7.463	7.622	0.159	3.890	−3.890	3.732	0.268	2.028
Au4-CME⊃ethylbenzene	−7.605	−0.162	7.443	7.605	0.162	3.883	−3.883	3.722	0.269	2.026

## Data Availability

Any additional data in support of the findings of this study besides those provided as Supplementary Materials are available from the corresponding author upon reasonable request.

## Supplementary Materials

The following Supporting Information can be downloaded at: https://www.mdpi.com/article/10.3390/molecules28196808/s1, Figure S1. Geometry-optimized structures: (a) Receptor molecule CME; (e) Receptor Au4-CME; 1:1 binding modes of (b) CME⊃benzene; (c) CME⊃toluene; (d) CME⊃ethylbenzene; (f) Au4-CME⊃benzene; (g) Au4-CME⊃toluene; and (h) Au4-CME⊃ethylbenzene; Figure S2. Comparison of the resonance frequency responses of the Au-coated QTFs (without functionalized calix[4]arene) with the different concentrations (10^−12^ M to 10^−6^ M) of each of the aqueous of aromatic hydrocarbons solution; Table S1. Calculated global scalar properties of benzene, toluene, ethylbenzene, calix[4]arene methoxy ester, Au4-calix[4]arene methoxy ester and it’s 1:1 complex in the gas phase; Table S2. Calculated global scalar properties of benzene, toluene, ethylbenzene, calix[4]arene methoxy ester, Au4-calix[4]arene methoxy ester and it’s 1:1 complex with analytes (benzene, toluene and ethylbenzene) at the CAM-B3LYP/LANL2DZ level of theory in water solvent system; Table S3. Calculated global scalar properties of benzene, toluene, ethylbenzene, calix[4]arene methoxy ester, Au4-calix[4]arene methoxy ester and it’s 1:1 complex with analytes (benzene, toluene and ethylbenzene) at the PBE0/LANL2DZ level of theory in the gas phase; Table S4. Calculated global scalar properties of benzene, toluene, ethylbenzene, calix[4]arene methoxy ester, Au4-calix[4]arene methoxy ester and it’s 1:1 complex with analytes (benzene, toluene and ethylbenzene) at the PBE0/LANL2DZ level of theory in water solvent system.
