# Supplementary material for: Detection of Aromatic Hydrocarbons in Aqueous Solutions Using Quartz Tuning Fork Sensors Modified with Calix[4]arene Methoxy Ester Self-Assembled Monolayers: Experimental and Density Functional Theory Study
Source: Molecules. 2023 Sep 26;28(19):6808. doi: 10.3390/molecules28196808 (PMC10574471; doi:10.3390/molecules28196808)
Supplement: Supplementary file 1 [file molecules-28-06808-s001.zip › molecules-2612684-supplementary.pdf]

Detection of Aromatic Hydrocarbons in Aqueous Solutions Using Quartz Tuning Fork Sensors Modified with Calix[4]arene Methoxy Ester Self-Assembled Monolayers: Experimental and Density Functional Theory Study

Shofiur Rahman [1,*], Mahmoud A. Al-Gawati [1,2], Fatimah S. Alfaifi [2], Wadha Khalaf Alenazi [2], Nahed Alarifi [2], Hamad Albrithen [1,2], Abdullah N. Alodhayb [1,2,*] and Paris E. Georghiou [3,*]

[1] Biological and Environmental Sensing Research Unit, King Abdullah Institute for Nanotechnology, King Saud University, P.O. Box 2455, Riyadh 11451, Saudi Arabia
[2] Department of Physics and Astronomy, College of Science, King Saud University, P.O. Box 2455, Riyadh 11451, Saudi Arabia; falfaifi@ksu.edu.sa (F.S.A.)
[3] Department of Chemistry, Memorial University of Newfoundland, St. John's, NL A1C 5S7, Canada
* Correspondence: mrahman1@ksu.edu.sa (S.R.); aalodhayb@ksu.edu.sa (A.N.A.); parisg@mun.ca (P.E.G.)

Table of Contents

	**Page**
**Figure S1.** Geometry-optimized structures: (**a**) Receptor molecule CME; (**e**) Receptor Au4-CME; 1:1 binding modes of (**b**) CME⊃benzene; (**c**) CME⊃toluene; (**d**) CME⊃ethylbenzene; (**f**) Au4-CME⊃benzene; (**g**) Au4-CME⊃toluene; and (**h**) Au4-CME⊃ethylbenzene	S3
**Figure S2.** Comparison of the resonance frequency responses of the Au-coated QTFs (without CME) with the different concentrations (10^{-12} M to 10^{-6} M) of each of the aqueous of aromatic hydrocarbons solution: (a) benzene, (b) toluene, (c) ethylbenzene, and (d-f) summary histogram showing the relative resonance frequency shifts (Δf) +/− ~ 5% to the three aromatic hydrocarbons tested with and without CME-functionalized Au-coated QTFs.	S4
**Table S1.** Calculated global scalar properties of benzene, toluene, ethylbenzene, CME, Au4-CME and their 1:1 complex computed at the CAM-B3LYP/LANL2DZ level of theory in the gas phase.	S5
**Table S2.** Calculated global scalar properties of benzene, toluene, ethylbenzene, CME, Au4-CME and their 1:1 complex computed at the CAM-B3LYP/LANL2DZ level of theory in the water solvent system.	S6
**Table S3**. Calculated global scalar properties of benzene, toluene, ethylbenzene, CME, Au4-CME and their 1:1 complex computed at the PBE0/LANL2DZ level of theory in the gas phase.	S7
**Table S4.** Calculated global scalar properties of benzene, toluene, ethylbenzene, CME, Au4-CME and their 1:1 complex computed at the PBE0/LANL2DZ level of theory in the water solvent system.	S8

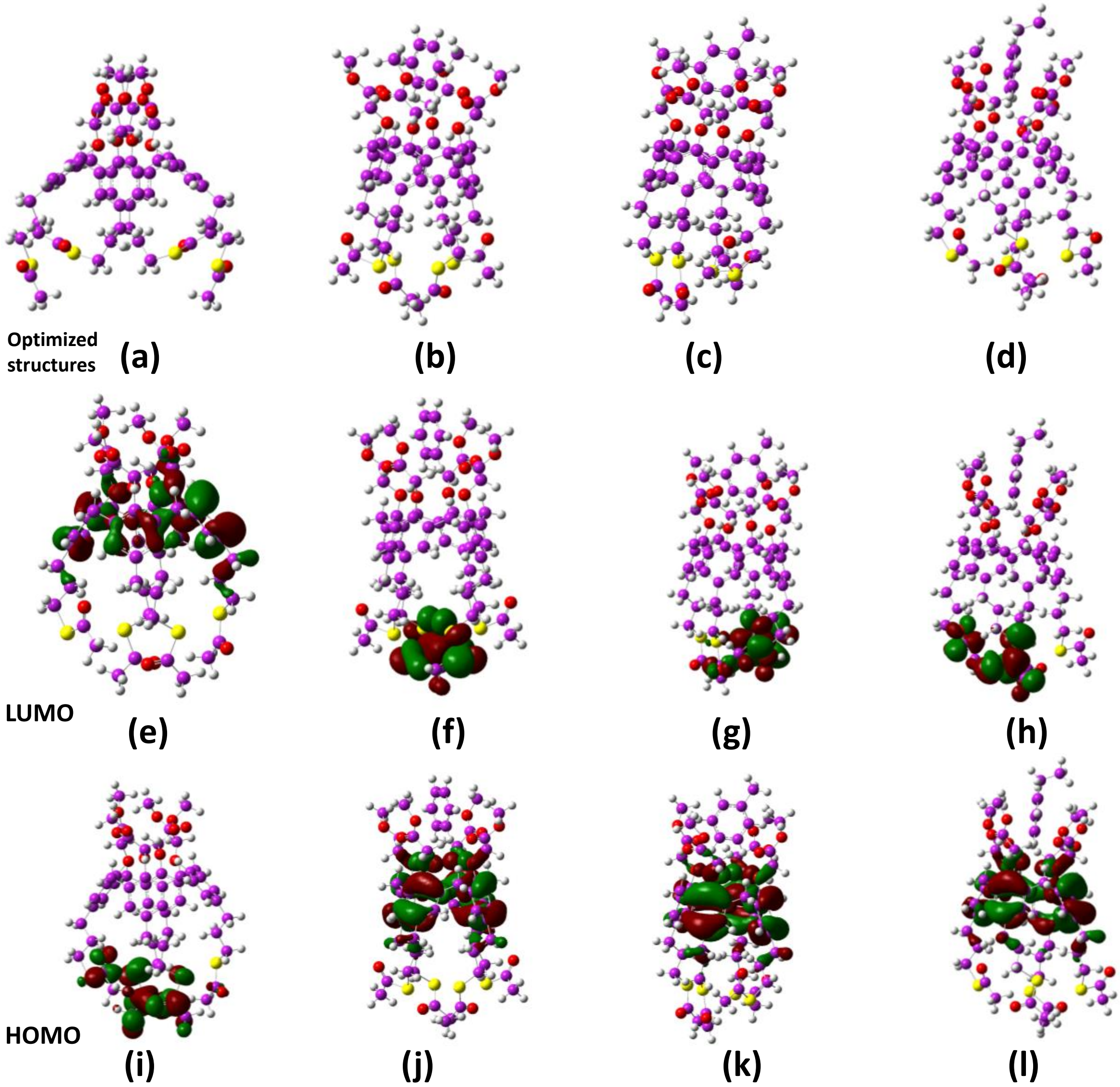

Figure S1. Geometry-optimized structures: (**a**) Receptor molecule CME; (**e**) Receptor Au4-CME; 1:1 binding modes of (**b**) CME⊃benzene; (**c**) CME⊃toluene; (**d**) CME⊃ethylbenzene; (**f**) Au4-CME⊃benzene; (**g**) Au4-CME⊃toluene; and (**h**) Au4-CME⊃ethylbenzene. Colour code: carbon = gray (except benzene, toluene, and ethylbenzene carbon = green); gold = orange; oxygen = red; sulfur = yellow; and hydrogen atoms = white for benzene, toluene, and ethylbenzene (other hydrogen atoms are omitted for clarity)..

Resonance frequency measurements:

Resonance frequency measurements from Au-coated QTFs functionalized without the calix[4]arene-methoxy ester (CME) sensing layer were used to quantitatively and qualitatively detect the presence of benzene, toluene, and ethylbenzene in water solutions. The response of the Au-coated QTFs without the CME shows very small frequency shifts in the order: benzene (35 Hz) > toluene (34 Hz) > ethylbenzene (31 Hz) as shown in Figure S2.

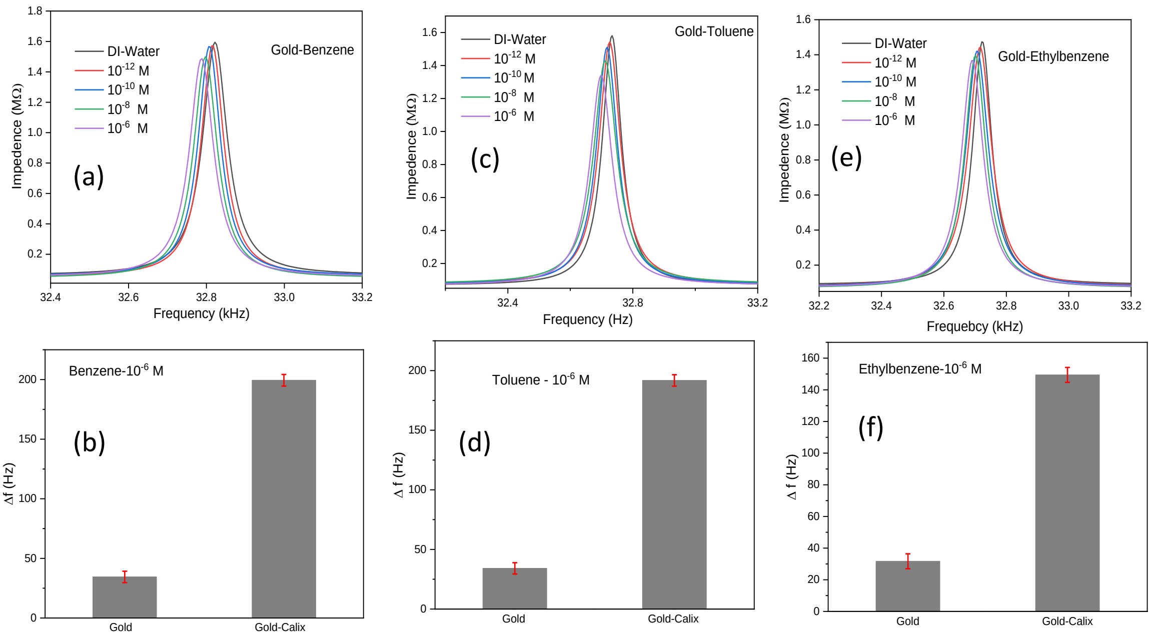

Figure S2. Comparison of the resonance frequency responses of the Au-coated QTFs (without CME) with the different concentrations (10^{-12} M to 10^{-6} M) of each of the aqueous of aromatic hydrocarbons solution: (a) benzene, (b) toluene, (c) ethylbenzene, and (d-f) summary histogram showing the relative resonance frequency shifts (Δf) +/– ~ 5% to the three aromatic hydrocarbons tested with and without CME-functionalized Au-coated QTFs.

Table S1 Calculated global scalar properties of benzene, toluene, ethylbenzene, CME**,** Au4-CME and their 1:1 complex computed at the CAM-B3LYP/LANL2DZ level of theory in the gas phase.

	**HOMO energy eV**	**LUMO energy eV**	**H-L gap eV**	**Ionization potential (IP) eV**	**Electron affinity (EA) eV**	**Electro-negativity (χ) eV**	**Chemical potential (μ) eV**	**Hardness (η) eV**	**Softness (S) eV**	**Electrophilicity Index (ω) eV**
Benzene	-8.364	-1.092	7.273	8.364	1.092	4.728	-4.728	3.636	0.275	3.074
Toluene	-6.741	-0.435	6.306	6.741	0.435	3.588	-3.588	3.153	0.317	2.041
Ethylbenzene	-6.750	-0.446	6.304	6.750	0.446	3.598	-3.598	3.152	0.317	2.054
CME	-7.027	-0.352	6.675	7.027	0.352	3.689	-3.689	3.337	0.300	2.039
Au4-CME	-7.230	-0.181	7.050	7.230	0.181	3.706	-3.706	3.525	0.284	1.948
CME⊃benzene	-7.150	-0.338	6.812	7.150	0.338	3.744	-3.744	3.406	0.294	2.058
CME⊃toluene	-7.124	-0.395	6.729	7.124	0.395	3.759	-3.759	3.364	0.297	2.100
CME⊃ethylbenzene	-7.171	-0.358	6.813	7.171	0.358	3.765	-3.765	3.407	0.294	2.080
Au4-CME⊃benzene	-7.337	-0.202	7.135	7.337	0.202	3.770	-3.770	3.568	0.280	1.992
Au4-CME⊃toluene	-7.325	-0.196	7.129	7.325	0.196	3.761	-3.761	3.564	0.281	1.984
Au4-CME⊃ethyl-benzene	-7.321	-0.195	7.126	7.321	0.195	3.758	-3.758	3.563	0.281	1.982

Table S2. Calculated global scalar properties of benzene, toluene, ethylbenzene, CME, Au4-CME and their 1:1 complex computed at the CAM-B3LYP/LANL2DZ level of theory in water solvent system.

	HOMO energy eV	LUMO energy eV	H-L gap eV	Ionization potential (IP) eV	Electron affinity (EA) eV	Electro-negativity (χ) eV	Chemical potential (μ) eV	Hardness (η) eV	Softness (S) eV	Electrophilicity Index (ω) eV
Benzene	-8.528	-0.920	7.609	8.528	0.920	4.724	-4.724	3.804	0.263	2.933
Toluene	-8.159	-0.938	7.221	8.159	0.938	4.549	-4.549	3.611	0.277	2.865
Ethylbenzene	-8.160	-0.980	7.179	8.160	0.980	4.570	-4.570	3.590	0.279	2.909
CME	-7.607	-0.268	7.338	7.607	0.268	3.937	-3.937	3.669	0.273	2.113
Au4-CME	-7.526	-0.206	7.319	7.526	0.206	3.866	-3.866	3.660	0.273	2.042
CME⊃benzene	-7.653	-0.226	7.427	7.653	0.226	3.939	-3.939	3.713	0.269	2.089
CME⊃toluene	-7.671	-0.189	7.482	7.671	0.189	3.930	-3.930	3.741	0.267	2.064
CME⊃ethylbenzene	-7.676	-0.144	7.532	7.676	0.144	3.910	-3.910	3.766	0.266	2.030
Au4-CME⊃benzene	-7.625	-0.160	7.465	7.625	0.160	3.892	-3.892	3.732	0.268	2.030
Au4-CME⊃toluene	-7.622	-0.159	7.463	7.622	0.159	3.890	-3.890	3.732	0.268	2.028
Au4-CME⊃ethyl-benzene	-7.605	-0.162	7.443	7.605	0.162	3.883	-3.883	3.722	0.269	2.026

Table S3. Calculated global scalar properties of benzene, toluene, ethylbenzene, CME, Au4-CME and their 1:1 complex computed at the PBE0/LANL2DZ level of theory in the gas phase.

	**HOMO energy eV**	**LUMO energy eV**	**H-L gap eV**	**Ionization potential (IP) eV**	**Electron affinity (EA) eV**	**Electro-negativity (χ) eV**	**Chemical potential (μ) eV**	**Hardness (η) eV**	**Softness (S) eV**	**Electrophilicity Index (ω) eV**
Benzene	-6.109	-0.991	5.118	6.109	0.991	3.550	-3.550	2.559	0.391	2.462
Toluene	-5.774	-0.912	4.862	5.774	0.912	3.343	-3.343	2.431	0.411	2.299
Ethylbenzene	-5.772	-0.900	4.872	5.772	0.900	3.336	-3.336	2.436	0.410	2.285
CME	-4.868	-1.857	3.010	4.868	1.857	3.363	-3.363	1.505	0.664	3.756
Au4-CME	-5.007	-1.838	3.168	5.007	1.838	3.423	-3.423	1.584	0.631	3.697
CME⊃benzene	-4.892	-1.937	2.954	4.892	1.937	3.414	-3.414	1.477	0.677	3.947
CME⊃toluene	-4.854	-1.930	2.923	4.854	1.930	3.392	-3.392	1.462	0.684	3.936
CME⊃ethylbenzene	-4.881	-1.927	2.954	4.881	1.927	3.404	-3.404	1.477	0.677	3.923
Au4-CME⊃benzene	-5.103	-1.838	3.264	5.103	1.838	3.471	-3.471	1.632	0.613	3.690
Au4-CME⊃toluene	-5.091	-1.832	3.259	5.091	1.832	3.462	-3.462	1.629	0.614	3.677
Au4-CME⊃ethyl-benzene	-5.056	-1.822	3.235	5.056	1.822	3.439	-3.439	1.617	0.618	3.657

Table S4. Calculated global scalar properties of benzene, toluene, ethylbenzene, CME, Au4-CME and their 1:1 complex computed at the PBE0/LANL2DZ level of theory in water solvent system.

	**HOMO energy eV**	**LUMO energy eV**	**H-L gap eV**	**Ionization potential (IP) eV**	**Electron affinity (EA) eV**	**Electro-negativity (χ) eV**	**Chemical potential (μ) eV**	**Hardness (η) eV**	**Softness (S) eV**	**Electrophilicity Index (ω) eV**
Benzene	-6.294	-1.159	5.135	6.294	1.159	3.726	-3.726	2.567	0.389	2.704
Toluene	-5.965	-1.107	4.858	5.965	1.107	3.536	-3.536	2.429	0.412	2.574
Ethylbenzene	-5.959	-1.096	4.863	5.959	1.096	3.527	-3.527	2.432	0.411	2.558
CME	-5.393	-2.049	3.344	5.393	2.049	3.721	-3.721	1.672	0.598	4.141
Au4-CME	-5.357	-2.029	3.328	5.357	2.029	3.693	-3.693	1.664	0.601	4.098
CME⊃benzene	-5.438	-2.104	3.334	5.438	2.104	3.771	-3.771	1.667	0.600	4.265
CME⊃toluene	-5.423	-2.091	3.332	5.423	2.091	3.757	-3.757	1.666	0.600	4.236
CME⊃ethylbenzene	-5.442	-2.039	3.403	5.442	2.039	3.740	-3.740	1.702	0.588	4.111
Au4-CME⊃benzene	-5.419	-2.027	3.392	5.419	2.027	3.723	-3.723	1.696	0.590	4.087
Au4-CME⊃toluene	-5.416	-2.030	3.387	5.416	2.030	3.723	-3.723	1.693	0.591	4.093
Au4-CME⊃ethyl-benzene	-5.417	-2.030	3.387	5.417	2.030	3.724	-3.724	1.693	0.591	4.094
